# An Intervention by and for Transgender Women Living With HIV: Study Protocol for a Two-Arm Randomized Controlled Trial Testing the Efficacy of “*Healthy Divas”* to Improve HIV Care Outcomes

**DOI:** 10.3389/frph.2021.665723

**Published:** 2021-10-27

**Authors:** Jae M. Sevelius, Torsten B. Neilands, Cathy J. Reback, Danielle Castro, Samantha E. Dilworth, Rachel L. Kaplan, Mallory O. Johnson

**Affiliations:** ^1^Department of Medicine, Center for AIDS Prevention Studies, University of California, San Francisco, San Francisco, CA, United States; ^2^Department of Medicine, Center of Excellence for Transgender Health, University of California, San Francisco, San Francisco, CA, United States; ^3^Friends Research Institute, Los Angeles, CA, United States; ^4^Center for HIV Identification, Prevention and Treatment Services, University of California, Los Angeles, Los Angeles, CA, United States; ^5^Department of Obstetrics, Gynecology, and Reproductive Sciences, Bixby Center for Global Reproductive Health, University of California, San Francisco, San Francisco, CA, United States

**Keywords:** transgender women, gender affirmation, health care empowerment, behavioral composite of engagement in HIV care, virologic control, HIV care engagement, two-arm randomized controlled superiority clinical trial

## Abstract

**Introduction:** Transgender women (assigned “male” at birth but who do not identify as male) are disproportionately impacted by HIV and experience unique barriers and facilitators to HIV care engagement. In formative work, we identified culturally specific and modifiable barriers to HIV treatment engagement among transgender women living with HIV (TWH), including prioritizing transition-related healthcare over HIV treatment, avoiding HIV care settings due to gender-related and HIV stigma, concerns about potential drug interactions with hormones, and inadequate social support. Grounded in the investigators' Models of Gender Affirmation and Health Care Empowerment, we developed the *Healthy Divas* intervention to optimize engagement in HIV care among TWH at risk for treatment failure and consequential morbidity, mortality, and onward transmission of HIV.

**Methods and Analysis:** We conducted a 2-arm randomized controlled trial (RCT) of the intervention's efficacy in Los Angeles and San Francisco to improve engagement in care among TWH (*N* = 278). The primary outcome was virologic control indicated by undetectable HIV-1 level (undetectability = < 20 copies/mL), at baseline and follow-up assessment for 12 months at 3-month intervals.

**Ethics and Dissemination:** This study was approved by University of California, San Francisco Institutional Review Board (15-17910) and Western Institutional Review Board (20181370). Participants provided informed consent before enrolment in the study. We are committed to collaboration with National Institutes of Health officials, other researchers, and health and social services communities for rapid dissemination of data and sharing of materials. The results will be published in peer-reviewed academic journals and scientific presentations. We will make our results available to researchers interested in transgender health to avoid unintentional duplication of research, as well as to others in health and social services communities, including HIV clinics, LGBT community-based organizations, and AIDS service organizations.

**Clinical Trial Registration:** Clinicaltrials.gov, identifier NCT03081559.

## Introduction

### Background and Rationale

Evidence of the disproportionate rates of HIV infection, AIDS-related mortality, and uncontrolled viral load among transgender women has been rapidly increasing. Transgender women have 49 times higher odds of HIV infection compared to other groups, a disparity that exists across cultures and socioeconomic boundaries (1). Disparate prevalence of HIV is particularly pronounced for African American transgender women (2). In San Francisco, high HIV incidence and prevalence persist among African American transgender women, at 1.4 per 100 person-years and more than 30%, respectively (3). Compared to other populations in the city, viral suppression is lowest among transgender women living with HIV (TWH) (68%) (4). Further, lack of engagement in care and not being on antiretroviral therapy (ART) is high among TWH in San Francisco at 14.3 and 13%, respectively (5). Among African American TWH who had ever enrolled in HIV care in nearby Alameda County, more than one third had not received ART (6). Similarly, in Los Angeles, HIV prevalence among transgender women is high at 16.7% (7). Although transgender individuals make up only 0.1% of the city's population, 1.3% of all people living with HIV and 1.4% of recently diagnosed individuals are transgender (7). Among African American people living with HIV in Los Angeles, 26.5% are transgender (7) and among street- and venue-recruited transgender women, overall HIV prevalence ranges from 21.9–26.1% (8, 9). Transgender women are estimated to have the lowest proportion of viral suppression of any behavioral risk group (69%) (10).

Active engagement in clinical care and high levels of ART adherence are critical to allow people with HIV to live longer, healthier lives. Among persons diagnosed with HIV infection, 63% have successful virologic control as evidenced by suppressed viral load (11). Inconsistent engagement and retention in care (i.e., defined as at least two HIV care visits within 12 months with each visit 3 months apart) has been associated with death among HIV+ adults (12). TWH are less likely to be retained in HIV care and receive ART than other groups, (13, 14) and TWH on ART demonstrate worse ART adherence (15) and viral suppression (16) than non-transgender people on ART and report less confidence in their abilities to integrate treatment regimens into their daily lives (17). Further, like other populations, TWH not on ART or are not adherent to ART are at increased risk for elevated viral load, which increases the risk of emergence of drug-resistant virus and transmission of HIV to others. Among TWH, experiencing higher levels of social adversity is significantly associated with lower odds of viral suppression (18), with African American/Black TWH less likely to be retained in care and to reach viral suppression than Black non-transgender counterparts (19).

Addressing the culturally unique barriers to HIV care and adherence is vital to improving health outcomes among TWH (20). TWH face a complex array of psychosocial challenges that complicate their access and adherence to HIV care, such as limited access to and refraining from healthcare due to stigma and past negative experiences with providers, prioritization of gender-related healthcare, and concerns about adverse interactions between ART medications and hormone therapy. (21, 22) Social and economic marginalization due to transphobia (negative societal attitudes toward transgender persons) often result in poverty and unstable housing, familial alienation, limited formal education, limited social support, mental illness, trauma and victimization, substance use, and introduction to sex work often at an early age (23–29). These factors can result in late or no presentation to HIV medical care and poor health outcomes (30). Culturally responsive intervention efforts, in addition to gender affirming services and caring relationships with interventionists (31), may be most effective for improving care engagement and health outcomes among TWH at the individual level (32). Recent evidence from Los Angeles and San Francisco indicate that among transgender women of color living with HIV, the implementation of peer-delivered interventions positively impacts HIV care visit attendance, receipt of ART prescriptions, and retention in HIV care (33–36).

The non-HIV related medical concerns of transgender women (regardless of HIV status) differ from non-transgender persons. The desire for gender-affirming healthcare, such as hormone therapy, is a critical factor that is sometimes at odds with effective engagement in HIV medical care (37). Anecdotal reports from healthcare providers indicate that hormone treatment can be an incentive for TWH to seek and adhere to ART (38). In our formative work, TWH repeatedly reported misinformed fears that ART can limit the effect of hormones, a serious concern for this population. Finally, in our prior work, TWH have reported that HIV medical providers often do not have expertise in transgender health and that providers specializing in transgender health do not also specialize in HIV care. When transgender women were compared to other respondents, regardless of the current medication regimen, they were significantly less likely to report positive interactions with healthcare providers (17). Taken together, TWH may feel lost between multiple medical settings that are not integrated. Faced with this dilemma, many TWH have reported that they may priorities gender-affirming healthcare over HIV treatment (39).

**Figure 1:**
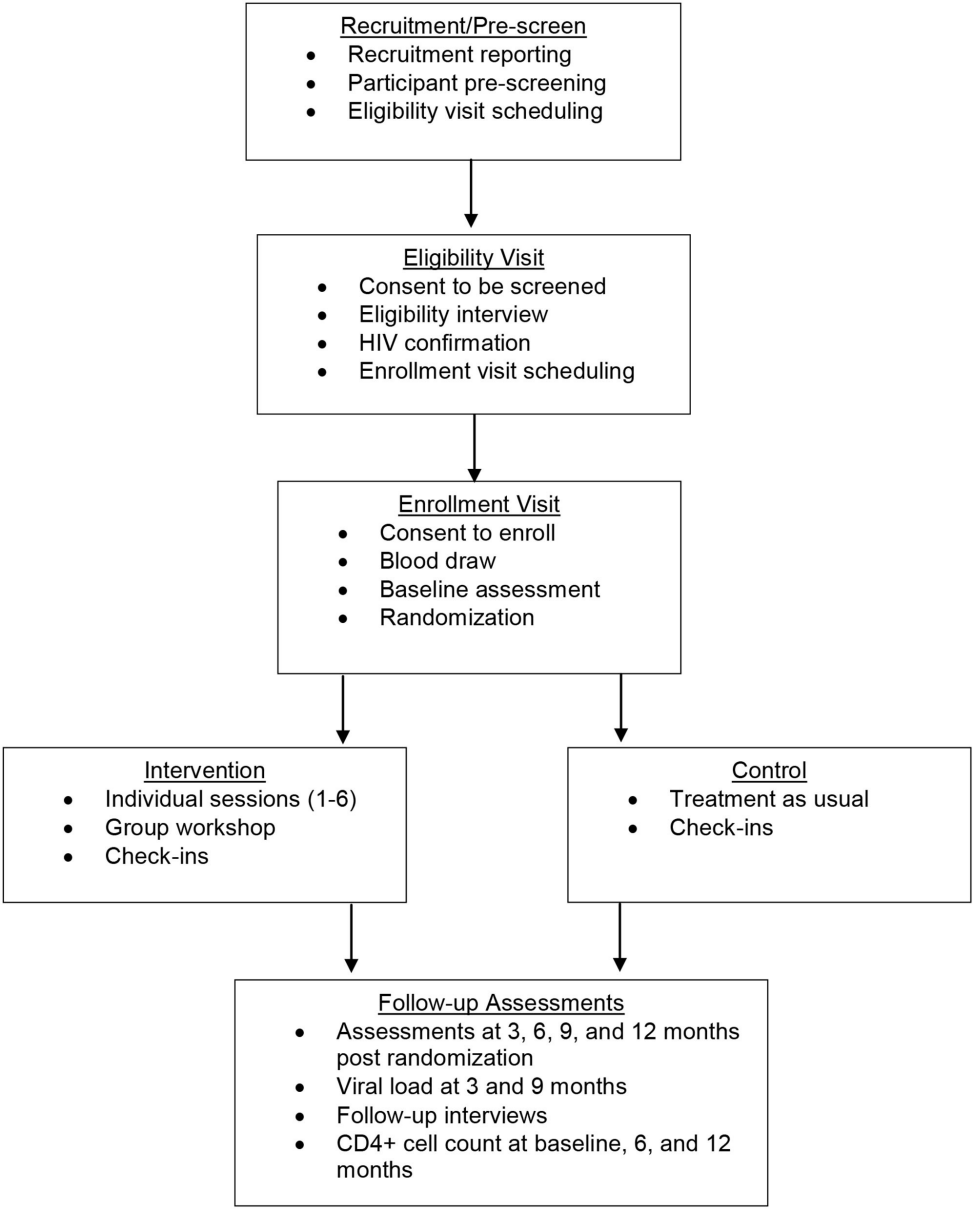
Schedule of enrollment, intervention, and assessments / CONSORT Diagram.

**Figure 2:**
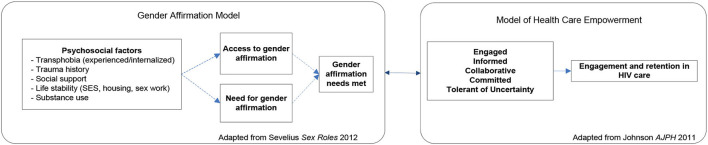
*Healthy Divas* conceptual model integrating the Models of Gender Affirmation and Health Care Empowerment.

This study is a randomized controlled trial (RCT) to intervene systematically on complex barriers to optimal engagement in HIV care specifically for TWH (see Figure 1 for Schedule of enrolment, intervention, and assessments / CONSORT Diagram). It is grounded in the investigators' Models of Health Care Empowerment (HCE) (40–42) and Gender Affirmation (GA) (43) (see Figure 2 for the *Healthy Divas* conceptual model integrating the Models of Gender Affirmation and Health Care Empowerment). We developed the *Healthy Divas* intervention to optimize engagement in HIV care for TWH at elevated risk for virologic failure and consequential morbidity, mortality, and transmission of HIV. We conducted a feasibility pilot test of the intervention with TWH using the same eligibility criteria as this RCT. We did not design the pilot to detect an effect size with which to power this RCT, as such efforts are problematic given the high likelihood of false negatives/positives inherent with small samples (44). Results from this RCT will have the potential to transform gender-affirming HIV healthcare for a population at dramatically elevated risk for disparately negative personal and public health outcomes.

#### Choice of Comparators

For our control condition, we considered several alternatives and weighed several issues, including Safer and Hugo's guidance for identifying active intervention elements while controlling for potential confounding effects (45). When considering a time and attention matched control condition, we found that identifying an inert intervention that would not directly or indirectly tap into the constructs of gender affirmation and healthcare empowerment through discussions of topics of interest was a challenge. Such a control intervention may have had non-specific effects that threatened our power to detect effects of the *Healthy Divas* intervention. Therefore, we decided on a treatment as usual (TAU) control condition in which participants randomized to the control condition were offered the intervention content after their final assessment point. As part of this design, we used measures to assess and control for exposure to adherence counselling and support outside of the trial for TWH in both conditions (46). We also accounted for variability in the treatment as usual control condition by assessing standard care quality (SCQ), as described by de Bruin et al., and used the SCQ measure as a covariate in our analyses of intervention effects. (46, 47) Recognizing that an effect on outcomes using this design will provide useful but incomplete evidence for this intervention, we identified potential future steps to evaluate the effectiveness of this intervention in comparison to other approaches. Given the relative strengths and weaknesses of any choice of control condition, our design represents a critical next step in the development of the evidence base for a high-risk population with disparately poor HIV outcomes.

### Objectives

Building from our formative quantitative and qualitative interviews with patients and providers, focus groups, and piloting and refinement of the intervention, this RCT had the following aims:

Primary Aim 1: To evaluate odds of virologic control for TWH in response to the *Healthy Divas* intervention.

Hypothesis 1: TWH randomized to *Healthy Divas* will achieve or maintain higher odds of undetectable viral load than TWH assigned to a treatment as usual control condition.

Secondary Aim 2: To evaluate the efficacy of the intervention on HIV treatment engagement among TWH.

Secondary Hypothesis 2: TWH assigned to *Healthy Divas* will have higher engagement in HIV care (appointment attendance, ART uptake, adherence, and persistence) than the control group.

Secondary Aim 3: To explore the effect of the intervention on hypothesized mechanisms of action.

Secondary Hypothesis 3: TWH assigned to *Healthy Divas* will report more favorable scores on theory-based constructs of healthcare empowerment, gender affirmation, adherence self-efficacy, satisfaction with medical care, social support, HIV treatment information, and treatment beliefs and expectancies.

### Trial Design

This study was designed as an interventional 2-arm randomized controlled superiority clinical trial with two parallel groups and a 1:1 allocation to compare *Healthy Divas* to a TAU control condition. The *Healthy Divas* intervention is a combination of six individual sessions with a peer counsellor and a peer-led small group workshop with other TWH, and a provider with expertise in HIV primary care and transgender healthcare, all completed within 3 months. TWH (*N* = 278) were enrolled at two sites (117 in San Francisco; 161 in Los Angeles) who were confirmed to be living with HIV and showed evidence of suboptimal engagement in care, defined as meeting one or more of the following three indicators: (a) not on ART, (b) on ART but reporting non-adherence, (c) reporting no HIV care appointments in the prior 6 months. The primary outcome was virologic control, defined as undetectable viral load. Secondary exploratory outcomes were (a) within-subject improvement in scores on our engagement in HIV clinical care composite index, which includes ART uptake, ART adherence, and self-reported attendance at HIV primary care appointments; and (b) change in hypothesized mechanisms of action (e.g., healthcare empowerment, gender affirmation). The intervention began immediately following randomization and was completed prior to the 3-month follow-up assessment. Follow-up assessments, including viral load assays and surveys, occurred for 12 months post-randomization at 3-month intervals (see Figure 1 for the Schedule of enrolment, intervention, and assessments). Monthly check-in visits promoted contact between visits and allowed regular monitoring of HIV medical care.

## Methods

### Participants, Interventions, and Outcomes

#### Study Setting

This study was conducted in San Francisco and Los Angeles, California. An estimated more than 900 transgender women live in the city of San Francisco (46, 47), not including hundreds of transgender women in the East Bay region. Epidemiological data from Los Angeles County estimates that there are over 7,000 transgender women living in Los Angeles County (DHSP, 2012). All study activities occurred in community-based research locations separate from clinical care sites, minimizing confounding with clinic attendance. In San Francisco, our field site was located in the Tenderloin area and in Los Angeles our field site was located on the border between Hollywood and West Hollywood, areas with high HIV prevalence and community viral load burdens in San Francisco and Los Angeles, respectively. Both sites have working relationships with dozens of community-based agencies and clinics in the area and are centrally located near public transportation access.

#### Eligibility Criteria

Potential participants were considered eligible if they were ≥18 years; assigned male sex at birth but did not currently identify as male; English- or Spanish-speaking; HIV-positive confirmed via antibody testing; and reported suboptimal engagement in HIV care, as indicated by one or more of the following: (a) Not on ART; (b) If on ART, reporting less than perfect adherence on a validated adherence rating scale; or (c) Reporting no HIV primary care appointments in the prior 6 months.

Potential participants were considered ineligible if they exhibited evidence of severe cognitive impairment or active psychosis, as determined by the Project Director in consultation with the Principal Investigator (PI), a licensed clinical psychologist.

Individuals were not excluded if they were actively engaged in substance use. Substance use is prevalent in TWH, and exclusion based on active substance use would have resulted in a sample that does not represent the target population. Informed by our prior work (21), the intervention includes a focus on the role of substance use and alcohol in optimizing engagement in care. However, if an individual was intoxicated at the time of a visit, we rescheduled the session to preserve the integrity of the data collection and intervention delivery.

We considered only enrolling TWH with detectable viremia but opted for inclusion based on indicators of poor engagement instead. This approach allowed the intervention to prevent potential treatment failure in TWH whose patterns of engagement in care put them at risk of poor outcomes. We contended that this was likely to be a more cost-effective long-term approach, as preventing virologic failure may have greater individual and public health benefit than waiting to intervene until treatment failure. It was also more exportable to community settings, thus enhancing external validity; if the intervention demonstrates efficacy, agencies will be able to adopt it with easier screening procedures than if eligibility had been based on clinical parameters such as viral load. From a methodological perspective, this decision allowed more statistical power, as the primary outcome could fluctuate in both directions for the two randomized groups, thereby increasing internal validity. We anticipated that 50% of the sample at baseline would have undetectable viral load.

#### Interventions

The *Healthy Divas* intervention was developed to address the unique HIV care needs of TWH by providing relevant information, support, and skills building for the identification and accomplishment of individualized healthcare goals related to both gender transition and HIV care and treatment. *Healthy Divas* consists of 6 peer-led individual sessions and one peer-led group workshop of 3–7 participants attended by a healthcare provider with expertise in HIV care and transgender health. As a group, participants have the opportunity to ask questions and receive accurate information and guidance to address barriers to engagement in healthcare. The intervention supports TWH in optimizing their health outcomes by increasing healthcare empowerment with a gender-specific and affirming approach. For example, in *Healthy Divas*, TWH build skills to cope with transphobia and HIV stigma, become active and collaborative in their treatment planning, and proactively address challenges to adherence and in their relationships with providers. *Healthy Divas* also incorporates transgender-specific concerns about substance use, provides opportunities for a participant to consider how substance use might be affecting her ability to reach her personal health goals, and supports her ability to access culturally competent treatment if she identifies this as a need.

The combination of individual sessions and a single group workshop permitted tailoring and flexibility to address the specific concerns of each participant while also capitalizing on a supportive group process with the presence and contribution of knowledgeable providers. Each intervention session was standardized through the use of a detailed facilitator manual specifying session content, procedures, exercises, and activities (see Table 1 for *Healthy Divas* intervention activities outline).

**Table 1:** *Healthy Divas* intervention activities outline.

**Topics**	**Participant activities**
**Session one**	
• Welcome and introduction • Assessment • Vision of the future • Introduction to goal setting • Setting health-related goals	• Overview of intervention • Discuss personal history and health care history • Assess understanding HIV treatment and adherence • Discuss substance use and impact on health and HIV • Identify vision of future healthy self • Goal setting basics • Recalling and amplifying a gender affirming experience
**Session two**	
• Strengths and resiliency • Workshop preparation	• Review goal • Discuss role personal strengths • Set attainable goals • Reviews vision of the future • Discuss expectations of workshop • Create a list of questions for providers at workshop • Recalling and amplifying a gender affirming experience
**Session three**	
• Workshop review • Communication • Communication and health	• Review goal • Feedback on experience of workshop • Discuss remaining questions and areas to clarify • Discuss communication with health care providers • Discuss impact of transphobia, stigma-related trauma • Learn about fight or flight response • Strategize to decrease distress re: provider visits • Rehearse communicating with provider • Recalling and amplifying a gender affirming experience
**Session four**	
• Support and health • Relationships/keeping you and the ones you love safe	• Review goal • Identify current sources of support • Discuss beliefs around autonomy • Identify impact of transphobia, stigma-related trauma • Identify current self-care strategies • Discuss substance use impact on self-care • Identify resources for support • Set goals to increase self-care and access • Recalling and amplifying a gender affirming experience
**Session five**	
• Recognizing and celebrating progress • Recognizing and addressing barriers to improving health	• Review goal • Identify successes and challenges • Problem-solves barriers to completing goals • Identify additional resources • Recalling and amplifying a gender affirming experience
**Sessions six**	
• Review of intervention • Planning for future health	• Review goal • Identify progress • Review skills learned in intervention • Revisit vision of future healthy self • Participant sets goals for future • Participant visualizes achieving goals
**Group workshop (to occur within 3 months)**	
• Gender care & HIV care • Treatment adherence • Provider relations	• Providers discuss gender health, hormone therapy, other gender related treatments and accessing care • Providers discuss HIV, medications and adherence • Q and A between participants and providers • Discuss maximizing relationships with health providers • Relaxation/ breath awareness exercise

Individual sessions with the peer counsellor used consistent opening content, discussion points, and methods for generating individualized content from the participant's unique circumstances. Individual sessions also employed skills training which, depending on the session's focus, include role-playing, behavior rehearsal or practice exercises, and communication problem-solving. The group workshop provided an opportunity for participants to share experiences, brainstorm solutions with each other and with a provider to barriers to accessing health care and to optimal adherence and ask questions and receive accurate information. Workshop topics were generated by the participants and tailored to address their concerns, and included information about hormones and HIV treatment, transphobia, experiences with healthcare, concerns about HIV treatment, and how to communicate effectively with providers. In summary, the *Healthy Divas* intervention includes specific issues relevant to TWH and is organized along the constructs of the models of GA and HCE (see Table 2 for Outcomes and measures).

**Table 2:** Outcomes and measures.

	**Domain**	**Measure (reference)**	**# items**	**α**
**Primary outcome**	Virologic control	COBAS® AmpliPrep/COBAS® TaqMan® HIV test kit (Roche Molecular Systems, Inc.); threshold for undetectability = < 20 copies/mL.	NA	NA
**Secondary outcomes and intervention mechanisms (mediators)**	Treatment engagement	Behavioral composite of engagement in HIV care:1. Current/Past ART use2. HIV appointments timeline follow back3. ART adherence (Lu et al. rating (48) and Walsh VAS) (49)4. Knowledge of current CD4 cell count	10	0.74–0.85
	Gender affirmation	Previously created in K08 research (e.g., current hormone use, access to trans-related health care)	22	0.74
	Health care empowerment	Two subscales (ICCE and TU) (40)	8	0.85–0.90
	HIV treatment knowledge/beliefs	Balfour HIV treatment knowledge (50)Beliefs about medications wuestionnaire (BMQ) (51)Consequences of non-adherence (52)	8 12 4	0.82–0.90
	Adherence self-efficacy	HIV-ASES (53)	12	0.74–0.90
	Treatment readiness	ART expectancies/readiness (54)	12	0.81–0.88
	Social support	Social support questionnaire (55)	6	0.94
	Coping	Coping self-efficacy scale (56)	13	0.80
	Patient communication	Patient communication index (57)	5	0.85
	Shared decision making	Autonomy preference index (58)Decision-making opportunity scale (59)Decisional balance (single item) (60)	6 3 1	0.82–0.84
**Background and potential moderators**	Background/demographic	Age, race, ethnicity, income, education, sex work, relationship status, housing stability, incarceration history, transgender-specific health care, CD4+ cell.	18	NA
	Experiences of transphobia	Experiences of transphobia (adapted from schedule of racist events) (61)	18	0.96
	Experiences of racism	schedule of racist events (61)	18	0.91
	History of trauma	Trauma history questionnaire (62)Recent trauma screening item (63)	24 1	0.85 NA
	Positive and negative affect	Depressive symptoms: CES-D scale (64)Positive / negative affect: differential Emotions scale (DES) (65, 66)	20 30	0.86–0.91
	HIV stigma	HIV Stigma Scale Sowell (67, 68)	12	0.86
	Substance use	DAST-10 (drug screening) (69)Alcohol use disorders identification test (AUDIT) (70)Drug use frequency (71, 72)	10 10 10	0.80–0.94
	Transgender identification	Adapted from multigroup ethnic identity measure (73)	13	0.82

Intervention delivery training included detailed review of the intervention approach, procedures, and research ethics. Population-specific training included how to address trans-specific issues and cultural issues specific to African American and Latina TWH. Specific techniques for adhering to a manualized intervention while allowing for flexibility to address individual participant needs were be taught, role-played and supplemented by instructional readings including one written by Co-I Johnson (74). Ongoing training provided updates on HIV treatment and weekly group supervision; individual supervision addressed cases and procedural issues in intervention implementation. In this RCT, we offered the study in both English and Spanish.

#### Outcomes

This RCT's primary outcome was virologic control as indicated by an undetectable HIV-1 level on the COBAS® AmpliPrep/COBAS® TaqMan® HIV test kit (Roche Molecular Systems, Inc.), which has a threshold for undetectability = < 20 copies/mL and was conducted at each 3-month assessment visit. The secondary outcome was a behavioral composite of engagement in HIV care scale (75), which integrates current/past ART use, HIV appointments timeline follow back, ART adherence (adherence rating (48) and visual analog scale (49)), and knowledge of current CD4 cell count.

We developed a behavioral composite of engagement in HIV care scale (75) that operationalizes key behavioral dimensions of HIV care engagement: attendance in HIV primary care and ART-related engagement, which includes uptake, adherence, and persistence with ART. Importantly, the composite was designed to measure changes in engagement in response to intervention. For this reason, there is not a separate scale for those on ART vs. not (see Table 3. Behavioral composite of engagement in HIV care scoring). Indicators are additive and, by design, include some overlap. For example, if Participant A tested HIV-positive 2 years ago and has had a primary care visit in the prior 6 months, she would get one point for that indicator. This would also, by definition, give her one point for a primary care visit attended in the prior 12 months (two points total so far). If there were two visits in the prior 12 months that were at least 3 months apart (the current HRSA definition of retention in care), she would get one additional point (three points total so far). If she reports knowing her CD4+ count from a recent test (one point), is taking ART (one point), and her adherence is <80% (no point) but she reports no lapses in ART coverage of > 4 days in the prior 3 months (one point), her total engagement score is six.

**Table 3:** Behavioral composite of engagement in HIV care scoring.

**Engagement indicator**	**Points**
Primary care appt within prior 12 months	+1
Primary care appt within prior 6 months	+1
Continuous care criterion for 12 months	+1
Knows recent (w/in 6 mos) CD4+ cell count	+1
On ART	+1
>80% adherence prior 30 days	+1
>90%adherence prior 30 days	+1
No lapses in ART >4 days in prior 3 months	+1
**Total possible score**	**8**

Another example is Participant B who tested HIV-positive 8 months prior, has never seen a provider for HIV care and thus does not know her CD4+ cell count and is not taking ART. She would score a zero on the index. The goal is to help Participant A increase her ART adherence and for Participant B to link to an HIV primary care provider. If successful, both of these participants would increase their scores on the composite index of engagement by at least one point. Thus, the goal of the intervention is to increase the individual's score on the index of engagement along the HIV care continuum. Favorable score changes on the index of engagement in care is a secondary outcome of this RCT and was hypothesized to be the main driver of improvements in viral suppression (our primary outcome). Using an abbreviated form of the index in one of our available datasets of 253 HIV-infected adults, we found that scores of 4–5 were associated with five-fold increased odds of virologic control and a score of six with a six-fold increase in odds of virologic control, compared to those scoring <4 on the index (see Table 4. Behavioral composite scores and virologic control). The eight-point index allows greater precision to distinguish between levels of engagement in care. We hypothesized that if TWH improve their index scores in response to the *Healthy Divas* intervention, they will increase their likelihood of virologic control.

**Table 4:** Behavioral composite scores and virologic control.

**Index of engagement score**	**% with virologic suppression**	**OR of virologic suppression**
<4 (referent)	15%	—
4–5	47%	5.16^*^
6	55%	6.29^*^

* *indicates a p-value of.05 or less; N = 254*.

#### Participant Timeline

From the date that we enrolled our first participant to the conclusion of the final participant's 12-month follow-up assessment, this RCT took 47 months to implement. Follow-up assessments, including viral load assays and surveys, occurred for 12 months post-randomization at 3-month intervals (For measures, see Table 2. Outcomes and measures). A 29-day window, defined as 14 days before and 15 days after the due date, was available for participants to complete follow-up assessments (Figure 1 provides a schematic diagram of the schedule of enrolment, intervention, control, and assessments). At the time of this manuscript submission, RCT analyses were starting, and we anticipate all data analyses and dissemination of primary trial results to conclude in 12 months.

#### Sample Size

Our sample size was selected to accommodate a range of 40–60% undetectable viral load at baseline, which we monitored in consultation with our Data Safety Monitoring Board (DSMB) to ensure enrolment of a sample with sufficient variability in viral load. Power analyses were generated using the two-group repeated proportions module in NCSS PASS 12 (76) to compute minimum detectable effect sizes at the proposed *N* and α = 0.05 and power = 0.80 for the primary analysis to evaluate Hypothesis 1 (TWH randomized to *Healthy Divas* will achieve or maintain higher odds of undetectable viral load than TWH assigned to a treatment as usual control condition). The study enrolled 278 participants evenly assigned to the intervention and control groups. Prior to starting implementation, we assumed 20% attrition, so that data from 228 participants would be available for analysis at all time points (i.e., 114 participants per group). We computed the minimum detectable odds ratio (*OR*), proportion difference (*pdiff*), and standardized proportion difference (*h*) for a time-averaged comparison, assuming four post-baseline measurements and assuming a range of virologic control base rates P_0_ and the within-subject correlation ρ was varied between.20 and.80 (see Table 5 for minimum detectable effect size estimates). Minimum detectable effect size estimates for our primary analyses fall between cutoffs of 0.20 and 0.50 for small and medium standardized effect sizes (77), respectively, suggesting that primary analyses have sufficient power to detect small to small-medium effects.

**Table 5:** Within-subject correlations.

	**P**_**0**_ **= 0.30**			**P**_**0**_ **= 0.55**			**P**_**0**_ **= 0.80**		
**ρ**	***OR***	***Pdiff***	***h***	***OR***	***Pdiff***	***h***	***OR***	***Pdiff***	***h***
0.20	1.64	11.2%	0.235	1.61	11.6%	0.234	1.93	8.5%	0.235
0.30	1.71	12.3%	0.257	1.68	12.7%	0.257	2.07	9.2%	0.258
0.40	1.78	13.2%	0.275	1.75	13.6%	0.276	2.21	9.8%	0.277
0.50	1.84	14.1%	0.293	1.81	14.5%	0.294	2.35	10.4%	0.297
0.60	1.91	15.0%	0.311	1.88	15.3%	0.311	2.50	10.9%	0.314
0.70	1.97	15.8%	0.327	1.95	16.1%	0.328	2.65	11.4%	0.332
0.80	2.03	16.6%	0.344	2.01	16.8%	0.343	2.82	11.8%	0.347

Sample size calculations described above to evaluate Hypothesis 1 were also used for Hypothesis 2 (TWH assigned to *Healthy Divas* will have higher engagement in HIV care) and Hypothesis 3 (TWH assigned to *Healthy Divas* will report more favorable scores on theory-based constructs of healthcare empowerment, gender affirmation, adherence self-efficacy, satisfaction with medical care, social support, HIV treatment information, and treatment beliefs and expectancies). For secondary mediation analyses to address the mediation component of Aim 2 (TWH assigned to *Healthy Divas* will have higher engagement in HIV care than the control group, which will lead to higher odds of virologic control), the R function *getn.logistic.bcs* (78) was used to compute the minimum detectable odds ratio for the indirect effect of the intervention on virologic control *via* a mediator for the worst case scenario depicted in Table 5, *P*_0_ = 0.80 and *OR* for the direct effect = 2.82. Assuming α = 0.05, power = 0.80, a minimum *N* = 228 balanced on a binary intervention arm predictor and a high correlation of 0.80 between the mediator and intervention predictor, the minimum detectable *OR* = 2.49, *pdiff* = 10.9%, and *h* = 0.313. Alternative scenarios with lower base rates, smaller direct effects, or lower predictor-mediator correlations would be able to detect smaller mediation effects at the same power.

#### Recruitment

Consistent with our prior work, we took a multi-pronged approach to recruitment: posting flyers in targeted areas; performing outreach to agencies, clinics, community-based organizations, shelters, single room occupancy hotels; and accepting direct peer and provider referrals. Recruitment was conducted by experienced teams of transgender field staff. All interviews and intervention procedures were conducted at trans-friendly field sites where many transgender women live or congregate. In San Francisco, our field site was located in the Tenderloin area, and in Los Angeles our field site was located on the border between Hollywood and West Hollywood, areas with high HIV prevalence and community viral load burdens in San Francisco and Los Angeles. Both sites had working relationships with dozens of community-based agencies and clinics in the area. These connections, along with input from our community advisory boards and our strong working relationships with local agencies that serve transgender women, were used in recruitment efforts to meet project timelines. The enrolment period was conducted in 34 months. Recruitment efforts were monitored by study investigators. Individuals were paid $10 for completing eligibility activities and $30 for completing enrolment procedures. For complete incentive information, see Table 6. *Healthy Divas* incentive schedule.

**Table 6:** *Healthy Divas* incentive schedule.

**Control**	***Healthy Divas*** **intervention**
$10 for completing the eligibility visit.	$10 for completing the eligibility visit.
$30 for completing all enrolment procedures.	$30 for completing all enrolment procedures.
$40 for completing 3, 6, 9, and 12 month follow up assessments.	$30 for completing each of the 6 individual sessions.
$10 for each check-in on months 2, 4, 5, 7, 8, 10, and 11.	$40 for attending the group workshop.
$40 for up to 4 successfully enrolled participants referred. $10 per enrolled referral.	$40 for completing 3, 6, 9, and 12 month follow up assessments.
	$10 for each check-in on months 2, 4, 5, 7, 8, 10, and 11.
	$40 for up to 4 successfully enrolled participants referred. $10 per enrolled referral.
Total possible: $310	Total possible: $530

#### Patient and Public Involvement

The development of the *Healthy Divas* intervention and the implementation of the RCT were informed by a Community Advisory Board comprised of transgender women living with HIV.

### Assignment of Interventions

#### Allocation

##### Sequence Generation

Participants were randomly assigned via stratified randomization with randomly permuted block sizes of 2, 4, and 6. There were eight strata based on site, age <40 years old vs. not, and binary race (Black vs. not for San Francisco and Latina vs. not for Los Angeles) with a 1:1 allocation to either *Healthy Divas* or control condition using a computerized secure and fraud-resistant procedure employed in our team's prior studies. Our experience with over 700 participants in three RCTs has indicated that this method is successful for achieving balance across demographic, mediating, and outcome variables.

##### Concealment Mechanism

Allocation concealment was ensured, as the randomization procedure did not release the randomization code (to participants or study staff) until the participant had been recruited into the RCT after all baseline measurements had been completed.

##### Implementation

The study statistician generated the randomization scheme using SAS v.9.3. The resulting allocation list was stored in the data collection software package, REDCap, and maintained by the statistician. The research team did not have access to the randomization scheme at any time. The next available randomization status from the appropriate strata for each individual participant was delivered to the research assistant at the time of randomization only by REDCap. The research assistants enrolled participants into their assigned study arms.

#### Masking

Masking was not used in this trial; therefore emergency unmasking was also not used in this trial. Outcome assessors were not masked in this RCT because they informed participants of their study condition assignment. Peer counsellors did not serve as outcome assessors.

### Data Collection, Management, Analysis

#### Data Collection Methods

Potential participants gave consent to be screened for eligibility. An eligibility survey was administered by study staff; confirmation that participants were living with HIV was achieved via evidence of one of the following: HIV ART medicine prescribed to the participant; other medical documentation; rapid HIV testing provided by study staff; having previously participated in another research study for people living with HIV at either study site; or contact with the participant's care provider through signing a release of medical information form. If found eligible and following consent to enroll in the study, participants had their blood drawn, completed the baseline survey, provided extensive locator information, and were randomized to either intervention or control group.

##### Primary Outcome

To determine whether TWH randomized to *Healthy Divas* will achieve or maintain higher rates of undetectable viral load than TWH assigned to a treatment as usual control condition, the primary outcome was assessed. This RCT's primary outcome was virologic control as indicated by an undetectable HIV-1 level (undetectability = < 20 copies/mL). Virologic control was conducted at baseline and at each follow-up assessment for 12 months post-randomization at 3-month intervals. A study phlebotomist performed peripheral venous blood sample collections. This process included: completing laboratory requisitions forms with collection date, time, and other relevant information; preparing two tubes for HIV-RNA; performing venipuncture and blood draw; spinning and freezing each specimen; placing each labelled specimen and requisition form in a shipment bag for pick up. Results were securely accessed through the Care360^TM^ platform in San Francisco and Foundation Laboratory in Los Angeles and entered and saved in a study password-protected Access form.

##### Secondary Outcomes

Secondary outcomes included a behavioral composite of engagement in HIV care scale (75), which integrates current/past ART use, HIV appointments timeline follow back, ART adherence (adherence rating (48) and visual analog scale (49)), and knowledge of current CD4 cell count, in addition to the intervention's hypothesized mechanisms of action. Surveys were administered at baseline and at each follow-up assessment for 12 months post-randomization at 3-month intervals using the audio computer-assisted self-interviewing (ACASI) system and REDCap. Data were collected and managed using REDCap electronic data capture tools at UCSF (79). REDCap (Research Electronic Data Capture) is a secure, web-based application designed to support data capture for research studies, providing: (1) an intuitive interface for validated data entry; (2) audit trails for tracking data manipulation and export procedures; (3) automated export procedures for seamless data downloads to common statistical packages; and (4) procedures for importing data from external sources. Research assistants set participants up to use the ACASI and REDCap systems for baseline and follow-up survey assessments. see Table 2 for a full list of the measures used, including scale names/sources, number of items, and Cronbach's alpha. Data quality was promoted through initial, follow-up, and ongoing training of staff members conducting assessments, in addition to regularly held quality assurance meetings.

#### Retention

Our retention plan, developed by our team, has successfully retained >90% of research cohorts per year, and we conservatively estimated 80% retention for the 12-month period (80, 81). The retention plan included: presence at a central location close to public transit; maintenance of detailed contact information; skilled staff trackers; a reliable monthly check-in program (see below); graduated incentive payments (see Table 6. *Healthy Divas* incentive schedule); and efforts to foster positive attachments to the study.

Each participant was scheduled for brief monthly check-in visits in months in which there was otherwise no scheduled visit. At this visit, which was highly flexible and could occur in person or by phone, we updated contact information, clarified the next study visit date and time, documented 30-day self-reported adherence and any changes in medication regimens, and recorded any provider visits that had occurred in the prior month. Participants were paid a small incentive for this 10-min visit ($10). For this study, each monthly check-in visit had the added benefit of documenting HIV care appointment attendance while the events were still recent, which improved precision of our self-reported retention in care measurement. This timeline follow-back procedure was used in our pilot with positive results. Staff members had previously reported information available to them to facilitate this data collection. For example, an interviewer was able to say, “The last time we spoke was on [date] and at that time you said you had seen Dr. [provider's name here] at [clinic or hospital name] on [date previously provided]. Since that visit with Dr. X, have you attended any appointments?” We also inquired about missed appointments (i.e., scheduled but not attended) during each time period.

#### Data Management

All data were password protected and backed up daily to an encrypted secure server. Electronic copies of the data were stored on a secure server and any paper copies were destroyed at the end of the study. Participants' identity and data were handled with highest levels of care and confidentiality. The exceptions to confidentiality were those defined by law and include a suspicion of child abuse, elder abuse, and threat of imminent action on suicidal or homicidal ideation. The information provided by participants is coded with a number to help protect privacy; the link listing names with numbers is kept in locked files. Only study staff had access to study files. Other entities who may access research data include UCSF Committee on Human Research; Western Institutional Review Board; National Institutes of Health (NIH); University of California; and Friends Research Institute. Reports and publications about the study will never refer to participants by name. All staff members were trained in procedures for maintaining confidentiality of participant information.

#### Statistical Methods

##### Analysis Population and Missing Data

For preliminary analyses and missing data, one-way frequency tables for all variables and measures of central tendency and variability for continuous variables will characterize the sample. These analyses will be stratified by randomization group (i.e., intervention vs. control) to check for imbalances. If the intervention and control groups are found to differ significantly at baseline on one or more covariates (e.g., on ART vs. not), we will use methods based on the Rubin causal model (e.g., propensity scores, targeted maximum likelihood estimation, double-robust estimation) to obtain the desired marginal effect estimates under the counterfactual assumption of balanced groups (82–86). In general, we will address incomplete data multiple imputation (MI) (87) because MI makes the relatively mild assumption that incomplete data arise from a conditionally random (MAR) mechanism rather than the completely random process (MCAR) assumed by *ad hoc* methods such as listwise data deletion (88). Auxiliary variables will be included in analyses involving incomplete data to help meet the MAR assumption (89, 90) and sensitivity analyses will be conducted with pattern-mixture models to assess the robustness of the MAR assumption (91). SAS (92) will be used to perform the proposed analyses.

##### Outcomes

We estimated based on our prior work with the population that 20% of the sample will not be on ART at baseline and thus would have detectable viral load. The remaining estimated 80% of the enrolled sample would report limited engagement in care as evidenced by low ART adherence or poor appointment attendance. Therefore, we conservatively estimated that at least 40% (and as many as 60%) will have detectable viral load at baseline. Those who meet eligibility criteria were at increased risk of virologic failure resulting from poor engagement in care. If our intervention is successful, a higher proportion of intervention participants will display virologic control relative to control group participants.

Therefore, we hypothesized that, following the intervention, the odds of undetectable viral load will be higher for intervention participants than for control participants (Hypothesis 1). Our primary interest was to estimate the marginal or population-average effect of intervention participation on each outcome rather than the effect for a hypothetical average subject. (93) Moreover, within-subject correlations among outcomes are considered nuisance parameters rather than quantities of interest to be modeled explicitly. Accordingly, generalized estimating equations (GEE) will be used to perform the proposed primary analysis, which is a planned time-averaged comparison of post-baseline measurements across the intervention and control groups to test primary Hypothesis 1. Alpha will be set at 0.05 for this planned comparison. Any additional *post-hoc* comparisons (e.g., paired comparisons of the control and intervention groups at each time point) will maintain a nominal alpha level of 0.05 through the use of simulation-based stepdown multiple comparison methods (94). Reflecting the binary nature of the virologic control outcome, a binomial distribution and logit link will be used for this analysis. Though GEE estimates are consistent even if the correlation structure is misspecified, GEE's statistical efficiency improves as the working correlation structure more closely approximates the actual correlation structure (95). Thus, working correlation structures suitable for the study's design will be considered (e.g., unstructured, AR (1), exchangeable, *M*-dependent) (96). The QIC statistic will be used to select the final working correlation structure (97). Robust Huber-White “sandwich” standard errors will be used to obtain correct inferences even if the chosen correlation structure remains slightly misspecified. GEE case deletion diagnostics (e.g., DFBetas, Cook's *D*) will be used to investigate whether influential cases are present; if such cases are found, results will be reported with and without influential cases included (98). As noted above, standard care quality (SCQ) will be included as a covariate in this analysis. In addition, randomization strata indicators will be included as covariates to obtain unbiased results (99). Additional covariates may be included if they improve QIC.

For the secondary outcome of HIV care engagement, we hypothesized that women assigned to *Healthy Divas* will have higher engagement in HIV care at follow-up than those assigned to the control condition as measured by appointment attendance and ART uptake, adherence and persistence (Hypothesis 2). We will use the same GEE methods described above to explore whether TWH in the *Healthy Divas* intervention group have higher HIV treatment engagement relative to those in the control group.

##### Additional Analyses

To explore the effect of the intervention on hypothesized mechanisms of action, secondary analyses will use GEE to evaluate whether TWH assigned to the intervention report higher post-intervention mean scores on theory-based constructs such as health care empowerment, gender affirmation, adherence self-efficacy, social support, HIV treatment information, and treatment beliefs and expectancies (Hypothesis 3). These analyses will also investigate whether these constructs mediate the relationship between intervention group assignment and virologic control. Statistical mediation will be assessed using the causal inference-based approach of Valeri and VanderWeele, which yields optimal estimates of indirect effects in the presence of binary outcomes and moderator-mediator interactions (100). In these analyses mediators will always temporally precede outcomes to uphold temporal ordering assumptions. For instance, mediators at 3 months will be considered for analyses involving virologic control at 9 months and mediators at 9 months will be considered for analyses involving virologic control measured at 12 months.

### Monitoring

#### Data Monitoring

Recruitment goals, missing data, and follow-up failures were monitored monthly throughout the trial.

##### Formal Committee

The study's data and safety monitoring board (DSMB), the “University of California, Los Angeles (UCLA) DSMB for Addiction Medicine,” is independent from the sponsor; members do not have any competing interests. Board members were chosen for their relevant expertise on study content, target populations, and methodologies. The roles of our DSMB include: reviewing analyses of outcome data and data safety to determine whether the trial should continue as originally designed, should be changed, or should be terminated based on these data; reviewing trial performance information, such as accrual information; determining whether and to whom results should be released prior to the reporting of the study results; reviewing reports of related studies to determine whether the monitored study needs to be changed or terminated; reviewing major proposed modifications to the study prior to their implementation; and providing study leadership with written summary information following board meetings.

##### Interim Analysis

No interim analyses took place during the trial.

#### Harms

All safety-related risks of harm were monitored routinely at the time of the assessment or intervention session. The security of confidential information was monitored regularly. Study staff were trained in asking questions about sensitive topics in a caring and non-threatening manner and stopping questioning at the first sign of discomfort or on request. Privacy, confidentiality, and disclosure comfort were emphasized in every session. All participants were reminded that they were not required to disclose their HIV status (or any other personal information) to anyone at any time. Group facilitators requested that participants agree to respect the privacy and confidentiality of other members of the group and to not disclose anyone's personal information to anyone outside of the group. Participants were informed that assessment responses are kept confidential and are not used against them in any manner, including for reasons of legal action or medical treatment. Study staff were trained to identify a participant who reports distress. For participants who report distress or suicidality, a protocol guided staff action, including steps to assess the level of distress, to obtain emergency contact information for clinical supervisors, and to obtain up-to-date phone numbers for crisis centers, hotlines, and referral agencies.

Study staff were trained to report breach of confidentiality risks incurred by participants to the Project Director, who in turn was trained to inform the PI. Any participant in need of treatment due to distress was referred for appropriate services after staff followed the participant distress protocol and informed the Project Director and PI. Finally, the PI was responsible for informing the DSMB chair, IRB through the IRB adverse event reporting procedure, and the sponsor Project Officer through immediate email of any life-threatening incidents and through annual reports of other incidents. The PI was prepared to take appropriate action to stop the study, release a participant from the study, or modify procedures to reduce and/or eliminate the abovementioned risks if they occurred at an unacceptable level.

#### Auditing

No trial audits were planned or conducted, but participating institutions had the authority to perform random audits of research protocols.

## Discussion

The *Healthy Divas* intervention and corresponding RCT protocols were developed with extensive formative work, including input from TWH, providers, and other community stakeholders. The forthcoming results will represent a critical step in the development of an intervention that specifically addresses the unique and wide-ranging challenges experienced by TWH, a group whose disproportionate rates of HIV infection and poor outcomes warrant focused efforts. This work fills a significant public health gap through the evaluation of a theory-driven, piloted, culturally tailored intervention to improve engagement in HIV care among TWH. The project was established within two novel and complementary theoretical models developed by the investigative team; the Model of Gender Affirmation and the Health Care Empowerment Model offered a rich foundation on which to build the *Healthy Divas* intervention. We also implemented a behavioral composite of engagement in HIV care scale, which is supported by preliminary data and offers clear direction for promoting greater engagement in HIV. This RCT tested an innovative engagement in HIV care intervention designed specifically for TWH. It, therefore, potentially represents an important contribution in providing an evidence-based approach to improving HIV clinical outcomes among this population at heightened risk for treatment failure and onward transmission of HIV. In addition to the individual-level intervention described here, structural interventions are urgently necessary to improve HIV care outcomes among TWH (101).

Although the results of this RCT are not yet available, the *Healthy Divas* intervention is already being replicated in three US cities: Birmingham, Alabama; Newark, New Jersey; and Oakland, California. Funded by the Health Resources and Services Administration (HRSA) / HIV/AIDS Bureau (HAB) Ryan White HIV/AIDS Program (U69HA31067; U90HA31099), *Healthy Divas* is being implemented for 4 years (August 2017–July 2021). We attribute this early replication to the urgent need for effective gender affirming and transgender-specific peer led interventions focused on HIV treatment engagement and outcomes among TWH. We anticipate that the results from this RCT will have the potential to transform gender-affirming HIV health care for a population at dramatically elevated risk for disparately negative personal and public health outcomes.

### Strengths and Limitations of This Study

- Our multidisciplinary team integrated support for improvement of engagement in HIV care with gender affirmation to develop the *Healthy Divas* intervention and achieve optimal outcomes, rather than requiring transgender women living with HIV to compartmentalize their needs for HIV and transition-related support and care.
- This RCT was implemented in two urban settings with large numbers of transgender women living with HIV, making replication and scale-up relatively straightforward in other urban areas of the US where HIV care and gender affirming services are available for transgender women.
- A limitation of the intervention approach is that many challenges that contribute to HIV-related disparities among transgender women include social, economic, and structural factors, which are beyond the scope of individual-level interventions; however, individual-level interventions are urgently needed to help transgender women living with HIV develop skills and coping resources for navigating existing systems.

## Ethics and Dissemination

### Research Ethics Approval

This study was reviewed and approved by the University of California, San Francisco Institutional Review Board (15-17910) and the Western Institutional Review Board (20181370).

### Protocol Amendments

All protocol modifications were reviewed and approved by NIH prior to implementation of amendments. All modifications approved by NIH were submitted to the University of California, San Francisco and the Western Institutional Review Boards. The Principal Investigator made the appropriate changes (e.g., to extend the study period) to the protocol via clinicaltrials.gov. Major changes that would have warranted re-consenting participants did not take place.

### Consent or Assent

Two consent processes took place; signed informed consent was conducted for the eligibility visit and the enrolment visit. After an individual was screened and expressed interest in participating in the study, she provided signed informed consent for the eligibility visit. Signed informed consent for enrolment took place at the baseline enrolment visit. Study staff members who conducted assessments (i.e., “assessors”) completed the consent procedures.

### Ancillary Studies

Participant data and biospecimens were collected solely for the purposes of this study; no ancillary studies were planned or conducted.

### Confidentiality

The following confidentiality protection steps were implemented: study staff participated in training, ongoing monitoring, and supervision to ensure understanding of the ethical issues involved in this research; only trained staff knew the name, identification number, and contact information of participants; consent forms were kept in locked files; personal identifiers linked to data were removed and replaced by code numbers in all records. Electronic copies of data were stored on a secure password-protected server. Paper copies of data will be destroyed at the end of the study.

### Declaration of Interests

There are no competing interests among the study investigators.

### Access to Data

All investigators have been given access to the cleaned data sets. Project data sets are housed in REDCap; all data sets are password protected. Study investigators have direct access to data originating from both study sites. To ensure confidentiality, any identifying participant information has been removed from data dispersed to project team members.

### Ancillary and Post-trial Care

During study enrolment, participants received referrals to emergency health and psychological services if needed. Participants received a brochure with information about culturally appropriate and transgender-sensitive health, psychological, and social services at enrolment. No provisions for post-trial care were planned or provided.

### Dissemination Policy

#### Trial Results

Following study completion and publication of primary reports, research data will be shared in accordance with NIH guidelines (http://grants.nih.gov/grants/policy/data_sharing/). As Principal Investigator, Dr. Sevelius shared information about this trial via timely registration and updates in ClinicalTrials.gov and will provide results in accordance with NIH policy. The results will be published and provided in peer-reviewed academic journals and scientific presentations at national conferences. We will make our results available to the community of researchers and general public interested in transgender health to avoid unintentional duplication of research, as well as to others in the health and social services community, including HIV clinics, LGBT community-based organizations, and AIDS service organizations.

#### Authorship

The Investigators of the study will follow International Committee of Medical Journal Editors guidelines for determining authorship eligibility and order. Final authorship decisions will be made by the Principal Investigator. No professional writers will be used.

#### Reproducible Research

We will share protocols and study forms in response to specific requests. Requests for study data will be evaluated on an individual basis, and de-identified study data will be made available as appropriate only after publication of all study outcome analyses.

## Ethics Statement

The studies involving human participants were reviewed and approved by University of California, San Francisco Institutional Review Board (15-17910), Western Institutional Review Board (20181370). The patients/participants provided their written informed consent to participate in this study.

## Author Contributions

JS and MJ conceived of the trial and designed the study. JS is PI and grant holder. TN was study statistician. CR was site PI in Los Angeles. DC contributed to the design of the intervention. SD was data manager. RK compiled the first draft of the study protocol manuscript. All authors contributed to the manuscript and consented to final publication.

## Funding

This work was supported by National Institute of Mental Health (NIMH) R01MH106373 and University of California, San Francisco. The funding source had no role in the design of this study, its execution, analyses, interpretation of the data, or decision to submit results.

## Conflict of Interest

The authors declare that the research was conducted in the absence of any commercial or financial relationships that could be construed as a potential conflict of interest.

## Publisher's Note

All claims expressed in this article are solely those of the authors and do not necessarily represent those of their affiliated organizations, or those of the publisher, the editors and the reviewers. Any product that may be evaluated in this article, or claim that may be made by its manufacturer, is not guaranteed or endorsed by the publisher.
